# The Impact of *Ganoderma lucidum* (Curtis) P. Karst. Supplementation on the Technological, Chemical, and Quality Parameters of Wheat Bread

**DOI:** 10.3390/foods13193101

**Published:** 2024-09-28

**Authors:** Paulina Łysakowska, Aldona Sobota, Anna Wirkijowska, Piotr Zarzycki, Agata Blicharz-Kania

**Affiliations:** 1 Department of Engineering and Cereal Technology, Faculty of Food Science and Biotechnology, University of Life Sciences in Lublin, Skromna Street, 20-704 Lublin, Poland; paulina.lysakowska@up.lublin.pl (P.Ł.); anna.wirkijowska@up.lublin.pl (A.W.); piotr.zarzycki@up.lublin.pl (P.Z.); 2 Department of Biological Bases of Food and Feed Technologies, Faculty of Production Engineering, University of Life Sciences in Lublin, Głęboka Street, 20-612 Lublin, Poland; agata.kania@up.lublin.pl

**Keywords:** functional foods, bakery product, medical mushrooms, Reishi, dietary fibre, antioxidant properties

## Abstract

This study explores the incorporation of *Ganoderma lucidum* (Curtis) P. Karst. (Reishi mushroom) into wheat bread to develop a functional food with enhanced nutritional value. Reishi powder was added to bread formulations at levels of 3%, 6%, 9%, and 12% to assess its effects on physicochemical, nutritional, and sensory properties. The 12% Reishi supplementation resulted in a twofold increase in total dietary fibre (from 7.21 g to 17.08 g per 100 g dry matter) and significant (*p* < 0.05) elevations in mineral content, particularly calcium (68%), iron (32%), and manganese (61.9%). Carbohydrate content decreased markedly by 27%, contributing to a 19.33% reduction in caloric value. Reishi addition improved bread yield and reduced baking losses, enhancing production efficiency. However, higher Reishi levels negatively impacted bread volume, possibly due to interference with gluten network formation. An increase in crumb moisture was observed, contributing to extended freshness. Sensory evaluation revealed that loaves of bread containing up to 6% Reishi were acceptable to consumers, whereas higher levels detrimentally affected flavour and aroma. Therefore, Reishi-enriched bread, particularly with 6% supplementation, presents a promising functional alternative to conventional wheat bread, optimising nutritional benefits while maintaining consumer acceptability.

## 1. Introduction

In recent years, there has been a growing interest in enriching traditional food products, such as wheat bread, with health-promoting components to meet the demands of consumers seeking functional foods. One promising approach in this regard is the incorporation of *Ganoderma lucidum* (Curtis) P. Karst., commonly known as Reishi or Lingzhi, into wheat bread. This mushroom, highly valued in traditional Chinese medicine for its health-enhancing properties, contains approximately 400 bioactive compounds, including polysaccharides such as beta-glucans and heteropolysaccharides (glucose, galactose, frucose, mannose, arabinose); triterpenoids such as ganoderic acid, lucidenic acid, ganoderol, and ganodermadiol; sterols like ergosterol and lanosterol; as well as micro- and macroelements such as selenium, potassium, and magnesium. The richness of these substances suggests a potentially wide range of health benefits [1]. Reishi primarily exhibits anticancer properties by enhancing the activity of NK (natural killer) cells and lymphocytes, which effectively support the immune system in combating cancer cells. Furthermore, this mushroom is known for its hepatoprotective and antioxidant properties, which manifest in reducing oxidative stress and promoting liver regeneration. Reishi also has potent immunomodulatory effects, increasing T-cell counts and improving immune responses, which is beneficial in the treatment of allergies and chronic diseases. Additionally, studies have confirmed its antidiabetic effects, which are reflected in the reduction in HbA1c levels in patients with type 2 diabetes [2,3,4,5]. The nutritional value of Reishi depends on cultivation conditions, the part of the mushroom used, extraction methods, and the country of origin, all of which influence the content of bioactive compounds and its biological activity. The macronutrient content of Reishi can vary depending on the growing and processing conditions: protein, 7–28.6%; lipids, 1.52–5.8%; carbohydrates, 5.41–63.27%; and dietary fibre, 7.77–76.81% [3,6,7]. Moreover, Reishi may positively affect gut microbiota health and potentially regulate cortisol levels, which could support the body’s adaptation to stress [7,8]. Additionally, research on the thermal processing of Reishi suggests that roasting dried fruiting bodies increases their antioxidant capacity and β-glucan availability, potentially enhancing their prebiotic properties and ability to neutralise free radicals [9]. Reishi also holds potential as a functional ingredient in the pharmaceutical and nutraceutical industries, particularly in the context of research on COVID-19 prevention [10]. Introducing Reishi into wheat bread may open new possibilities for the development of bakery products that address contemporary dietary and environmental challenges while offering consumers potential health benefits [11]. Wheat bread, a dietary staple worldwide, not only provides energy but also serves as an important source of carbohydrates, dietary fibre, B vitamins, and mineral elements. Its global consumption underscores its widespread importance and crucial role in human nutrition [12]. The history of wheat bread, which spread globally and became a key element of the human diet, is closely linked to the domestication and development of bread wheat (*Triticum aestivum*), a process that began around 8500–9000 years ago [13]. The hybridisation and evolution of wheat have led to today’s diversity of bread types, which are tailored to different dietary preferences and nutritional needs. In response to these challenges, the bakery industry is turning to innovations, such as enriching wheat bread with health-promoting ingredients, among which *Ganoderma lucidum* presents itself as a promising additive. The presence of polysaccharides, triterpenoids, sterols, and micro- and macroelements in Reishi could contribute to the enhanced nutritional value of wheat bread while simultaneously offering health benefits [2,3,5]. Reishi’s adaptogenic properties, supporting the body’s adaptation to stress, may further contribute to improving consumers’ overall health. In light of challenges related to bread waste and the need to increase its health benefits, the use of innovative ingredients like Reishi may represent a crucial step in optimising bread production.

There is a lack of reports in the literature regarding the fortification of traditional wheat bread with Reishi powder. Previous research has largely concentrated on the use of Reishi extracts, which have been shown to reduce baking losses, with lower concentrations proving acceptable from a sensory standpoint [7]. However, the incorporation of ground *Ganoderma lucidum* fruiting bodies into bread remains insufficiently studied. Consequently, this investigation, which explores the addition of Reishi powder, is particularly significant as it fills this gap in the existing body of knowledge. It also provides important insights into the effects of whole Reishi fruiting bodies on the quality parameters, physicochemical properties, sensory attributes, and antioxidant potential of bread. The results could facilitate the wider application of medicinal mushrooms in the bakery sector.

## 2. Materials and Methods

### 2.1. Materials

For this study, type 750 wheat flour produced by Polskie Młyny (Warsaw, Poland) was used. The characteristics of this flour included the following: ash content at 0.74% dry matter, wet gluten content of 27.5% ± 1.0, gluten index 99.0 ± 0.3, falling number of 304 s ± 6, and an average particle size of 0.12 mm. As an additive for the bread, powdered Reishi mushroom (*Ganoderma lucidum*) (NatVita, Mirków, Poland) was used. Both raw materials were stored in dark, airtight containers at temperatures below 25 °C and a relative humidity of 60–65%. These storage conditions were maintained prior to both the baking process and the chemical analysis of the raw materials and bread.

### 2.2. Farinographic Characteristics of Dough

Various farinographic parameters of type 750 wheat flour and wheat flour with the addition of 3%, 6%, 9%, and 12% Reishi mushroom powder were analysed. The examined parameters included dough development time (DDT), water absorption (WA), dough stability time (ST), dough softening degree (DS), and farinograph quality number (FQN). Measurements were taken using a Farinograph-E device (Brabender, model 8110142, Duisburg, Germany) following the AACC 54-21 procedure [14]. Each parameter was measured three times for each sample.

### 2.3. Bread Production Process

Five different bread formulations were prepared, including a loaf of control bread (CON) made from 100% wheat flour and loaves of bread in which the wheat flour was replaced with 3%, 6%, 9%, and 12% Reishi powder, labelled in the experimental model as BR3, BR6, BR9, and BR12, respectively. The complete bread recipe included 600 g of wheat flour or a blend of wheat flour and Reishi powder, 9 g (1.5%) of table salt, 18 g (3%) of compressed yeast (*Saccharomyces cerevisiae*), and an amount of water precisely determined based on the water absorption (WA) measured at a consistency of 500 Brabender units. This preparation method allowed for the precise monitoring of the impact of ingredient proportions on the final bread characteristics. The methodology described by Wirkijowska et al. (2020) [15] was employed for the bread preparation. The dough-making process followed a single-phase method. First, the dough ingredients were combined in a BEAR Varimixer Teddy 5 L (Varimixer A/S, Copenhagen, Denmark) for approximately 3 min at low mixer speed, followed by an increase in speed, with the dough mixed for a farinographically determined development time to ensure full gluten network development. Fermentation was carried out in a proofer (Tefi Klima, pro 100, Debag, Bautzen, Germany) at a temperature of 30 °C and relative humidity of 85 ± 2% for 90 min. After 60 min, intermediate punching of the dough was performed, and the dough was then divided into three portions weighing 290 g ± 5 g. The portions were hand-shaped and placed in baking tins measuring 18 × 7.5 × 7.0 cm. The dough was left to proof for 30 min in the proofer at 30 °C and 85 ± 2% relative humidity. The fermentation time was monitored for each sample. The fermented dough was then baked in a baking oven (Helios, pro 100, Debag, Germany) at 230 °C for 30 min. After baking, the loaves were cooled for one hour at room temperature and then individually packed in polyethylene bags and stored at room temperature (20 °C, 50% humidity) prior to quality evaluation. This procedure ensured the reliability of the research process and a comprehensive analysis of the bread’s characteristics.

### 2.4. Evaluation of Bread Quality

Five hours after cooling, the bread’s characteristics were analysed, including bread yield, calculated using Equation (1); mass loss, determined using Equation (2); bread volume, measured by mustard seed displacement according to AACC method 10-05.010; specific volume (cm^3^/100 g), calculated as the ratio of bread volume to its weight; and crumb moisture content, determined according to AACC method 44-15.02. All analyses were performed in triplicate.

(1) $$BY=\frac{W_{1}}{W_{2}}\times 100\%$$

(2) $$BL=\frac{W_{3}-W_{1}}{W_{3}}\times 100\%$$

where *BY* denotes the bread yield, *W*_1_ represents the mass of the baked bread (measured one hour after removal from the oven), *W*_2_ is the mass of the flour used for the specific loaf of bread, *BL* indicates the mass loss during baking, and *W*_3_ refers to the mass of the dough weighed immediately before placing it in the oven.

### 2.5. Bread Porosity

To analyse the porosity of the bread crumb, a VHX-7000N digital microscope (Keyence Corporation, Osaka, Japan) was employed at a magnification of ×20–100. The porous structure analysis involved evaluating the number, size, and uniformity of pores within the internal structure of the bread. The microscope facilitated the acquisition of detailed images of the sample. The number of pores was counted based on images taken from randomly selected areas of the crumb (3 × 3 cm), allowing for an assessment of the bread’s aerated structure. The pore size was measured by determining the diameters of individual voids, providing insights into the uniform distribution of gases during fermentation and baking. Additionally, the uniformity of pore distribution across the sample was analysed, which was crucial for evaluating the consistency of the crumb’s structure. The porosity measurement results served as a significant indicator of bread quality, enabling an assessment of its texture and consistency.

### 2.6. Evaluation of Bread Colour Parameters

The bread colour parameters were evaluated following the methodology described by Wirkijowska et al. (2023) [16]. The colour of the bread crumb was measured using a spherical spectrophotometer (Chroma Meter CR-5, Konica Minolta, Sakai, Osaka, Japan), and the L*, a*, and b* values were expressed in the CIE Lab colour space. The L* value represents the lightness of the colour (ranging from 0 to 100, from black to white), the a* value indicates redness with positive values and greenness with negative values, and the b* value represents yellowness with positive values and blueness with negative values. The spectrophotometer was calibrated using standard black and white plates, and measurements were performed 10 times for each sample. The total colour difference (ΔE*) between the control sample and the enriched bread loaves was calculated according to Equation (3). Additionally, for each bread sample, the whiteness index (WI), yellowness index (YI), and browning index (BI) were calculated based on the L*, a*, and b* values using Equations (4), (5), and (6), respectively.

L_c_*, a_c_*, and b_c_* represent the values for the control sample (CON); L_i_*, a_i_*, and b_i_* represent the values for the samples enriched with Reishi mushroom. WI is the whiteness index, YI is the yellowness index, and BI is the browning index.

(3) $$\Delta E^{*}=\sqrt{{\left(L_{c}^{*}-L_{i}^{*}\right)}^{2}+{\left(a_{c}^{*}-a_{i}^{*}\right)}^{2}+{\left(b_{c}^{*}-b_{i}^{*}\right)}^{2}}$$

(4) $$\mathrm{WI}=100-\sqrt{((100-L^{*}{)}^{2}+a^{2}+b^{2}}$$

(5) $$\mathrm{YI}\;=\;142.83\cdot \frac{b^{*}}{L^{*}}$$

(6) $$\mathrm{BI}=\frac{100(x-0.31)}{0.17}\;x=\frac{\left(a^{*}+1.75L\right)}{\left(5.645L^{*}+a^{*}-{0.012b}^{*}\right)}$$

where

$L_{c}^{*},\;a_{c}^{*}$, $b_{c}^{*}$—the values for the control sample (CON);
$L_{i}^{*},\;a_{i}^{*}$, $b_{i}^{*}$—the values for tested samples enriched with (LM) mushroom;
WI—whiteness index;
YI—yellowness index;
BI—browning index.

### 2.7. Texture Profile Analysis (TPA) of Bread

The top sections of the bread loaves were removed, and each loaf was sliced into 20 mm thick slices. From these slices, cuboid-shaped samples measuring 30 × 30 × 20 mm were cut for further analysis. A double compression test was conducted using a Zwick/Roell Z0.5 strength testing machine (BT1-FR0.5TN.D14, ULM, Germany) with a maximum force of 500 N. The parameters included compression to 50% of the initial height, maintaining a constant head speed of 1 mm/s, and using a flat cylindrical disk with a diameter of 100 mm. The force–deformation curves we obtained were used to determine various properties such as hardness [N], springiness [-], cohesiveness [-], and chewiness [N]. The texture profile analysis (TPA) was conducted 24 and 48 h after baking, and each sample was measured seven times.

### 2.8. Chemical Analysis of Raw Materials and Bread

Detailed chemical analyses of the raw materials and bread were conducted, encompassing moisture, ash, protein, fat, and dietary fibre content, following AACC and AOAC methods [17,18].

For moisture analysis, 3 g samples were dried according to the AACC 44-15A method in a laboratory oven at a constant temperature of 103 °C ± 1 °C until a constant mass was achieved. Ash content was determined using the AACC 08-01 method. Samples weighing 3 g were placed in porcelain crucibles and incinerated in a muffle furnace at 550 °C for 7 h. After cooling, the samples were weighed, and the ash content was calculated.

The total protein content was determined using a Kjeltec™ 8400 apparatus (Foss Analytical AB, Höganäs, Sweden) with an automatic Kjeltec Auto device from Tecator. Nitrogen content was converted to protein using a conversion factor of N × 5.7.

The total fat content was determined following acid hydrolysis, followed by continuous extraction using a Soxtec™ 8000 apparatus (Foss Analytical AB, Höganäs, Sweden) with hexane as the solvent. The analyses were performed in triplicate to ensure the reliability of the results. Enzymatic methods (AACC 32-05, AACC 32-21, AOAC 991.43, AOAC 985.29) were used to determine the total dietary fibre (TDF), insoluble dietary fibre (IDF), and soluble dietary fibre (SDF). This process involved sequential enzymatic digestion of the dry samples (1 g) using thermostable α-amylase, protease, and amyloglucosidase. The enzymes and analytical procedures were developed by Megazyme International Ireland Ltd. (Wicklow, Ireland).

Digestible carbohydrates were calculated as the difference between 100% and the content of water, protein, fat, dietary fibre, and ash (USDA). The energy value was measured in kilocalories (kcal) per 100 g of fresh bread using Atwater factors. Proteins and carbohydrates provided 4 kcal/g, fats provided 9 kcal/g, and total dietary fibre was calculated at 2 kcal/g. This comprehensive analysis ensured a thorough evaluation of the bread’s composition and nutritional value.

### 2.9. Determination of the Content of Mineral Elements

In this study, the content of Ca, K, Fe, Cu, Mn, Pb, and Cd was determined using flame atomic absorption spectrometry (FAAS), in accordance with the PN-EN ISO 6869:2002 standard. Phosphorus was measured spectrophotometrically using a Shimadzu UV-1800 spectrophotometer.

### 2.10. Extraction of Polyphenols from Raw Materials and Bread

Polyphenol extraction from the raw materials and bread was carried out using 70% ethanol. Ten grams of each material was mixed with 90 mL of ethanol and heated in a water bath for 10 h at 40 °C, following the methodology described by Kozłowska et al. (2015) [19]. Filtration was then performed to separate the material from the solvent using filter paper. Fresh extracts were used to determine the total polyphenol and flavonoid content, as well as antioxidant capacities.

### 2.11. Total Polyphenol and Flavonoid Content in Raw Materials and Bread

The total polyphenol and flavonoid content were determined according to the methodology outlined by Krawęcka et al., (2022) [20]. In brief, the polyphenol content was measured using the Singleton and Rossi method with Folin–Ciocalteu reagent. A 0.1 mL sample was mixed with the reagent and 20% (*w*/*w*) sodium carbonate, and then it was incubated for 30 min. Absorbance was measured at a wavelength of 700 nm using a Thermo Spectronic Helios Epsilon (Thermo Electron, Waltham, MA, USA). Gallic acid (1–150 mg/L) was used as the standard, and the results were expressed as gallic acid equivalents (GAE) in mg/g of the sample.

The total flavonoid content was determined using the method proposed by Quettier-Deleu et al., (2000) [21]. A 2 mL sample of the extract was mixed with a 5% (*w*/*w*) aluminium chloride solution, and absorbance was measured at 405 nm after 30 min. Quercetin (0.25–20 mg/L) was used as the standard, and the results were expressed as quercetin equivalents (QE) in µg/g of the sample.

### 2.12. Antioxidant Activity of Raw Materials and Bread against DPPH· and ABTS^·+^

A DPPH ethanolic solution with a concentration of 6 × 10^−5^ mol/dm^3^ was prepared and diluted with ethanol to achieve an absorbance value of A = 0.70 at a wavelength of λ = 515 nm. The DPPH solution was stored in a dark place at room temperature. To 1.8 mL of the methanolic DPPH solution at a concentration of 6 × 10^−5^ mol/dm^3^, 100 μL of the test solution was added and thoroughly mixed. After 30 min, the absorbance was measured at a wavelength of λ = 515 nm. Ethanol was used as a control instead of the test sample. The entire analysis was repeated three times. The radical scavenging activity (RSA) was expressed as the percentage of DPPH neutralisation.

RSA (%) = (1 − At/A0) × 100(7)

where A0 is the absorbance of the control sample, and At is the absorbance of the test sample after 30 min.

Precisely 0.1920 g of ABTS and 0.0343 g of potassium persulfate were accurately weighed and quantitatively transferred into a 50 mL volumetric flask. The solution was stored in a dark place for 16 h. After this period, the ABTS solution was diluted with methanol to achieve an absorbance of A = 0.75 at a wavelength of λ = 734 nm. Subsequently, 100 μL of the test solution was added to 1.8 mL of the ABTS solution at a concentration of 6 × 10^−5^ mol/dm^3^ and thoroughly mixed. Changes in the concentration of ABTS cation radicals were determined spectrophotometrically after 30 min of incubation with the test extracts. After 30 min, the absorbance was measured at a wavelength of 734 nm. Water was used as a control instead of the test solution. The analysis was performed in triplicate. The radical scavenging activity (RSA) was expressed as the percentage of ABTS radical neutralisation.

RSA (%) = (1 − At/A0) × 100(8)

where A0 is the absorbance of the control sample, and At is the absorbance of the test sample after 30 min.

### 2.13. Sensory Analysis

The sensory analysis of the bread was conducted using a five-point scale. The evaluation followed established standards for bread quality assessment, in accordance with ISO 8586:2012 [22] guidelines for sensory analysis. The sensory panel consisted of eight trained assessors, selected based on their regular consumption of bread, good health, and absence of gluten allergies. Panellists were provided with detailed descriptors for each score on the scale, ensuring a clear understanding of what each rating represented. For example, the lowest score indicated significant flaws in the attribute being assessed, while the highest score represented optimal quality with no discernible defects, with intermediate scores reflecting varying degrees of quality. The assessors evaluated several sensory attributes. The external appearance was assessed based on crust characteristics, surface uniformity, and the crumb structure visible on a 1 cm thick slice. Aroma was evaluated immediately upon receiving the sample to capture the freshness of the bread. Elasticity was measured by gently pressing the crumb to observe its ability to return to its original shape. Porosity was assessed visually and through light tactile examination, considering the size and distribution of air pockets within the crumb. Taste was evaluated after chewing the sample, with attention to flavour intensity and aftertaste. Bread samples were sliced into 1 cm thick pieces using a mechanical bread slicer and labelled with random codes to ensure blind evaluation. The samples were presented in random order to prevent bias. The sensory analysis was conducted in a controlled sensory evaluation laboratory, with regulated lighting, temperature, and humidity conditions, in accordance with ISO 8589:2007 [23] standards, providing optimal conditions for objective assessment. The overall evaluation was calculated as the average of all individual scores for the assessed attributes. The study was approved by the Bioethics Committee (Resolution No. UKE/09/2023).

### 2.14. Statistical Analysis

The collected data were subjected to comprehensive statistical analysis. Mean values and their respective standard deviations were calculated to summarise the dataset. Statistical significance was determined using a one-way repeated measures analysis of variance (ANOVA). Post hoc comparisons were carried out using Tukey’s test. The analysis was conducted with the use of STATISTICA 13 software (StatSoft), with the significance level set at *p* ≤ 0.05.

## 3. Results and Discussion

### 3.1. Farinographic Properties of the Dough

In recent years, Reishi has gained popularity in the context of functional foods due to its bioactive components, such as polysaccharides, triterpenoids, and proteins, which influence the rheology and structure of bread dough by modifying its properties during baking [10]. The farinographic analysis of bread dough supplemented with *Ganoderma lucidum* revealed a significant impact of this addition on the dough’s rheological properties (Table 1). The control sample (CON) reached optimal consistency after 2 min and 30 s, which served as a reference point for further comparisons. The addition of Reishi at various concentrations (3%, 6%, 9%, 12%—designated as BR3, BR6, BR9, BR12) significantly affected parameters such as dough development time (DDT), water absorption (WA), stability time (ST), and degree of softening (DS). It was found that Reishi concentrations in the range of 3–9% significantly (*p* ≤ 0.05) shortened the DDT. This is consistent with the role of polysaccharides in modifying the rheological properties of food, as indicated by Wang et al. (2024) [24]. Polysaccharides, acting as plasticisers, increase dough’s water retention capacity, affecting its viscosity and elasticity while facilitating the formation of the protein network. At lower concentrations, the hydrophilic groups of Reishi polysaccharides promote gluten protein hydration, which in turn enhances dough elasticity and reduces the time required for the development of the protein network. In the case of the highest Reishi concentration (12%, BR12), the DDT was significantly prolonged (*p* ≤ 0.05). This suggests inhibition of key protein bond formation, likely due to the blockage of active protein sites by polysaccharides or triterpenoids present in Reishi, resulting in a reduced ability of gluten to form stable protein networks [25]. Additionally, excessive protein hydration caused by the high polysaccharide content increases dough viscosity and delays the network formation process. Water absorption (WA) systematically increased with rising Reishi concentrations, reaching the highest value of 63.3% in BR12. This is consistent with the hydrophilic nature of the polysaccharides in the mushroom, which enhance the dough’s water-binding capacity [26]. The high dietary fibre content, particularly insoluble fibre (IDF) in Reishi, may also contribute to the increased water absorption in the dough. Such hydration is critical for bread quality, as higher hydration during dough formation results in a moister crumb, making the bread softer and prolonging its freshness. Dough stability time (ST) showed a positive correlation with Reishi concentration, increasing from 6 min and 3 s in the control sample to 19 min and 8 s in BR12. Higher Reishi concentrations strengthened the dough structure, likely due to interactions between mushroom polysaccharides and gluten proteins. Concurrently, the degree of softening (DS) decreased as the Reishi concentration increased, suggesting that the mushroom component helps maintain the integrity of the gluten network and reduces its susceptibility to mechanical damage. The literature confirms that the formation of protein–polysaccharide complexes significantly influences dough’s hydration properties and gluten network stability. The high polysaccharide content in Reishi can lead to excessive gluten protein hydration, increasing dough viscosity and delaying gluten network formation, which is particularly evident in the sample with the highest (12%) Reishi addition. This sample exhibited a significantly longer development time (10:48) compared to the other samples [27].

### 3.2. Evaluation of Bread Quality Characteristics

**Figure 1:**
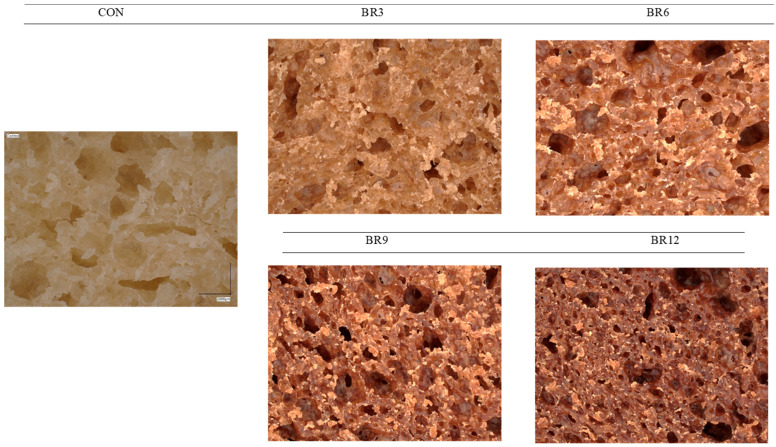
Porosity of fresh crumb of wheat bread (CON) and bread enriched with 3, 6, 9, and 12% Reishi (BR).

*Ganoderma lucidum*, an additive to wheat bread and a rich source of dietary fibre and polysaccharides, significantly affects its physical properties. Despite its beneficial impact on water retention and reduction in baking losses, higher concentrations of Reishi lead to a reduction in bread volume. This phenomenon can be explained by the disruption of the gluten network structure by the dietary fibre and polysaccharides present in Reishi, which limit gluten’s ability to trap air, resulting in a smaller loaf volume. Consequently, the dough’s ability to retain fermentation gases decreases, ultimately leading to a reduction in bread volume. Additionally, interactions between fibre and the gluten structure may further disrupt the integrity of the gluten network, directly impacting the final bread volume. The dietary fibre’s water-binding capacity also contributes to reduced baking losses by limiting moisture loss during the baking process [28]. Polysaccharides extracted from *Lycium barbarum* leaves, including both crude and degraded forms, had a significant impact on the quality of fresh bread dough, as confirmed through analyses of water-holding capacity, gluten structure, texture, and sensory acceptability. Electrophoretic studies demonstrated that crude polysaccharides destabilised gliadin, while degraded polysaccharides protected gluten, thereby improving dough texture. The application of these polysaccharides enhanced the dough’s extensibility properties and increased sensory acceptability, confirming their potential for improving the quality of bakery products [29]. Similarly, research by Guowei et al., (2019) [30] confirms the positive effect of *Ganoderma lucidum* on the quality of Chinese steamed bread, demonstrating that the addition of Reishi fermentation broth modified the microstructure of starch and proteins, which led to the expansion of starch granules and the enlargement of pores in the crumb, thereby improving bread volume. These findings suggest that enzymes, such as amylases, break down starch and catalyse protein cross-linking, further enhancing the texture and structural quality of the product [29,30]. These findings, summarised in Table 2, indicate that the polysaccharides present in Reishi, similar to those from mulberry leaves, can positively influence the structure and quality of bread [31]. Reishi, as a source of polysaccharides, can affect dough and bread properties by modulating the structure of starch and proteins. Both polysaccharides—from mulberry leaves and Reishi—exhibit the ability to modify bread microstructure, potentially leading to increased water retention and delayed staling processes. At the same time, the presence of dietary fibre and polysaccharides in Reishi reduces the integrity of the gluten network, which may result in decreased bread volume. Microscopic analyses of the bread crumb (Figure 1) illustrate changes in porosity depending on the addition of Reishi. In the control sample, a well-developed pore network with relatively large and regular dimensions was observed. As the Reishi concentration increased (from 3% to 12%), a reduction in pore size and irregularity became evident, indicating a denser and less aerated crumb structure. The reduction in porosity is most pronounced in the sample with 12% Reishi addition, suggesting that this supplement significantly affects the dough’s rheological properties, reduces its porosity, and results in a more compact bread texture. Optimising the amount of Reishi added to bread seems advisable to achieve bread with favourable physical properties while simultaneously enriching it with valuable nutrients.

### 3.3. Evaluation of Bread Colour Parameters

**Figure 2:**
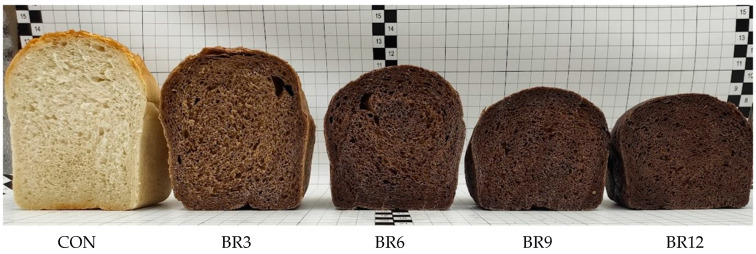
Samples of wheat bread (CON) and bread (BR) enriched with 3, 6, 9, and 12% Reishi.

The brownish-brown colour of Reishi (*Ganoderma lucidum*) is primarily attributed to the presence of terpenoids, melanin, and carotenoids, including β-carotene and lycopene, which together give the mushroom its characteristic hue. The carotenoid content in Reishi is approximately 4.47 mg/g, with β-carotene at 3.63 mg/g and lycopene at 0.224 mg/g, further contributing to its intense colour. To date, over 300 different triterpenoids have been isolated from this mushroom [32,33,34]. Triterpenoids are responsible for the brown or reddish hue, melanin contributes to the darker colour, and carotenoids influence the orange and red tones. Environmental conditions, such as light and temperature, can further modify these colours, consistent with observations of the colour variability of Reishi [35]. The addition of Reishi to bread significantly affects its sensory properties, particularly colour, which is a key parameter in assessing the quality of food products. Studies show that the addition of Reishi significantly (*p* ≤ 0.05) decreases the L* value (lightness), from 61.49 in the CON to 22.60 in the sample with 12% Reishi (Table 3). The a* value (green–red) increases from 0.51 in the CON to 9.29 in BS12, showing an intensification of the red hue with increasing Reishi content. Meanwhile, the b* value (blue–yellow) initially rises and then declines, suggesting complex changes in the yellow tones. The recorded colour changes were determined by an increase in the browning and yellowness indexes and a decrease in the whiteness index of the samples as a function of the increasing share of Reishi in the bread (Table 3). These results suggest that increasing concentrations of Reishi cause significant changes in bread colour parameters, leading to darkening, the intensification of the red hue, and increased browning of the samples. These changes of colour parameters are consistent with the known effects of bioactive compounds found in Reishi, such as melanin and terpenoids, on the colour of food products. In Figure 2, five bread crumb samples are shown. The control sample (on the left) exhibits a light colour typical of traditional wheat bread. As the concentration of Reishi increases (3%, 6%, 9%, and 12%), progressive darkening of the crumb is observed, which is a direct result of the natural pigments contained in Reishi. The intensity of the colour is proportional to the amount of Reishi added, indicating its significant impact on the colour of the final bakery product. Similar conclusions were presented by Cacak-Pietrzak et al., (2021) [36] in studies on bread enriched with dandelion roots (*Taraxacum officinale*), where increasing the additive amount led to the darkening of the bread, an increase in the intensity of the red colour, and a decrease in yellow intensity. It is worth noting that higher temperatures can further modify the colour intensity by reducing the L* value and altering the a* and b* parameters [37].

### 3.4. Texture Profile Analysis (TPA) of Bread

The analysis of bread texture is a key component in evaluating its quality and has a direct impact on consumer acceptance and product shelf life [38]. Studies have shown that the addition of Reishi mushroom significantly influences bread hardness, which increases proportionally with higher concentrations of this ingredient (Table 4). It is suggested that this effect is related to interactions between the bioactive compounds in Reishi and gluten proteins, leading to the strengthening of the gluten network. This reinforcement results in a less porous, more compact, and rigid dough structure, which in turn translates to increased bread hardness. Similar interactions that affect bread hardness also influence its springiness, chewiness, and cohesiveness. Springiness, defined as the ability of bread to return to its original shape after pressure is removed, exhibited a downward trend with higher Reishi concentrations. The stiffening of the gluten network, caused by the addition of Reishi, limits the flexibility of the dough matrix, leading to reduced springiness. The increase in hardness and decrease in elasticity also contributed to higher chewiness, meaning more effort was required during biting and chewing. This is a direct consequence of the structural changes in the gluten network, which also affect other textural properties. Cohesiveness, reflecting the bread’s ability to maintain its structural integrity, decreased as the Reishi concentration increased, potentially due to disruptions in the gluten network caused by the presence of insoluble dietary fibre components in Reishi. Similar effects were observed in studies examining the addition of mushroom powders such as button, shiitake, and porcini mushrooms to wheat bread. These additions resulted in increased hardness and decreased springiness and loaf volume, suggesting the possibility of enriching bread with dietary fibre and protein, but also indicating potential sensory drawbacks at higher substitution levels [39,40]. However, it should be noted that these results partially contradict observations from other studies on the impact of different mushrooms on bread texture. For example, the addition of mycelium from mushrooms such as *Antrodia camphorata*, *Agaricus blazei*, *Hericium erinaceus*, and *Phellinus linteus* showed the opposite effect—reducing hardness and improving bread springiness [40]. After 48 h, significant changes in the texture of the bread were observed, which can be attributed to the physico-chemical interactions affecting the samples. In terms of crumb hardness, a general increase in this parameter was noted across all samples. A particularly notable rise was recorded for sample BR6, where hardness increased from 7.70 N to 12.90 N. The highest hardness value was achieved by sample BR12, remaining at approximately 30 N, with changes between 24 and 48 h being insignificant, suggesting the structure of this sample had stabilised after 24 h.

The crumb springiness remained relatively stable in all samples, showing no statistically significant differences between 24 and 48 h. This indicates that the bread’s elasticity did not undergo major changes during the test period. Chewiness values, on the other hand, tended to increase, particularly in sample BR6, where they rose from 3.99 N to 5.20 N. This may suggest that the increase in crumb hardness contributes to greater resistance during chewing, thus affecting the overall texture of the product.

These differences may stem from the varying mechanisms of action of the bioactive compounds found in these mushrooms, which may also increase the elasticity of the gluten network in the dough, thereby enhancing crumb porosity and creating a softer bread texture.

### 3.5. Sensory Evaluation of Bread Characteristics

**Figure 3:**
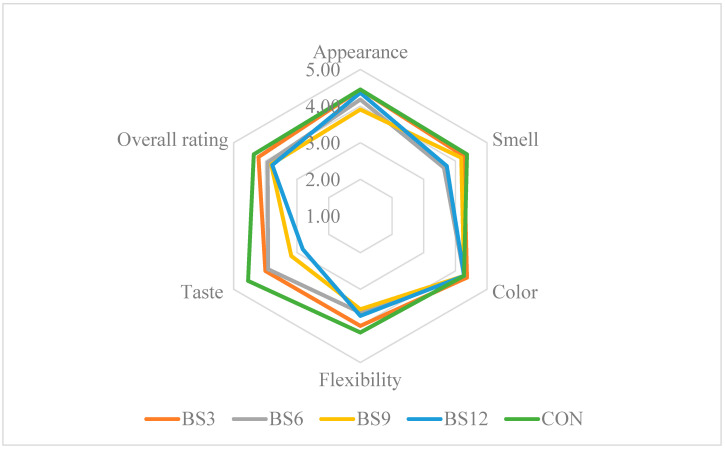
Results of the sensory evaluation of the bread. CON—control sample (100% wheat bread); BR—bread with 3, 6, 9, and 12% addition of Reishi.

The sensory evaluation of bread is a key tool in the process of product improvement, enabling a detailed analysis of consumer perception regarding attributes such as appearance, aroma, taste, texture, and overall product assessment [12]. In the context of introducing innovative ingredients to food, including medicinal mushrooms, sensory analysis plays a crucial role in assessing consumer acceptance of new products. Studies on bread enriched with various concentrations of Reishi mushroom powder (3–6%) revealed that small additions do not negatively affect attributes such as appearance, aroma, taste, and elasticity, with the evaluation results for these attributes being comparable to the control bread (Figure 3). In contrast, higher concentrations (9–12%) led to a significant deterioration in taste and aroma, suggesting that the intense aroma and flavour of the mushrooms may limit the product’s acceptance by consumers. Research by Gaglio et al., (2019) [41] showed that the addition of *Pleurotus eryngii* powder increased the intensity of the crust and crumb colour and influenced the bread’s aroma and flavour, while negatively affecting the porosity and elasticity of the crumb. Nevertheless, the overall sensory evaluation of bread with up to 10% *Pleurotus eryngii* powder indicated an acceptable sensory profile. Similar results were obtained in other studies on bread enriched with *Pleurotus eryngii* and *Cantharellus cibarius* powder. Although the addition of these mushrooms improved the bread’s nutritional value, it also led to a deterioration in some sensory attributes, particularly volume and aroma [42]. In contrast, the sensory analysis of gluten-free bread enriched with chaga (*Inonotus obliquus*) powder revealed that consumers most preferred samples with a low content of this ingredient (up to 10%). Higher concentrations (15% and 20%) significantly reduced the sensory ratings, particularly in the categories of taste, texture, and overall acceptance, which was attributed to the intense flavour and very dark colour of the bread [43]. These findings suggest that selected mushrooms, including Reishi, have considerable potential as functional additives to bread. When used in lower concentrations, they do not cause the significant deterioration of sensory attributes while serving as a source of bioactive compounds with multiple beneficial effects on the body. It is worth noting that the careful selection of mushroom quantities in the process of bread fortification is crucial for maintaining a balance between health benefits and consumer acceptance.

### 3.6. Chemical Composition of Raw Materials and Bread

The nutritional value of the raw materials and bread enriched with powdered Reishi mushroom is presented in Table 5. The introduction of Reishi into bread at various concentrations (3%, 6%, 9%, and 12%) significantly (*p* ≤ 0.05) influenced the content of selected chemical components, including ash, protein, dietary fibre, and digestible carbohydrates. An analysis of type 750 wheat flour, the reference raw material, showed a relatively low protein content (8.79% d.m.) and high digestible carbohydrate content (71.04% d.m.), which are the main sources of energy in wheat bread. However, the dietary fibre content in refined wheat flour was only 5.3% d.m. Reishi powder exhibits a significantly higher dietary fibre content (72.80% d.m.), with a predominance of the insoluble fraction (68.22% d.m.). The addition of Reishi powder to bread at higher concentrations contributed to a gradual increase in dietary fibre content, reaching 17.08% in bread with 12% Reishi, compared to 7.21% in the control bread. The increase in fibre, both insoluble (IDF) and soluble (SDF), is particularly important in the context of regulating gastrointestinal function and reducing the risk of lifestyle-related diseases such as obesity, type 2 diabetes, and atherosclerosis [43,44]. Insoluble fibre positively affects intestinal transit, helps prevent constipation, and increases the feeling of satiety after meals, while soluble fibre contributes to lowering blood cholesterol levels, which is crucial for cardiovascular health [45]. The protein content in Reishi-enriched bread BR12 increased by approximately 31.8% compared to the control bread. Although Reishi powder is characterised by low protein content, its presence may support protein metabolism through its bioactive compounds. Moreover, the diverse amino acid profile of the proteins present in *Ganoderma lucidum*, such as glutamate (1133.20 mg/100 g), aspartic acid (916.47 mg/100 g), and leucine (718.36 mg/100 g), may have a significant impact on their bioavailability, which is particularly important for individuals following vegetarian or vegan diets [10,46]. The digestible carbohydrate content in Reishi-enriched bread BR12 was significantly lower compared to the control bread, translating to a reduction of approximately 27%. Products enriched with Reishi provide a better alternative to typical wheat bread for individuals with metabolic disorders, such as obesity or type 2 diabetes [47]. The reduction in carbohydrate content, coupled with the increase in dietary fibre, contributes to a lower glycaemic index (GI) of the bread, promoting better glycaemic control after meals [48]. The caloric value of bread enriched with Reishi powder was also lower compared to the control bread. The addition of 12% *Ganoderma lucidum* resulted in a 19.33% reduction in the caloric content of the bread compared to the control. Similarly, the addition of mushroom powders (e.g., *Agaricus bisporus*, *Lentinula edodes*, *Boletus edulis*) to bread led to an increase in protein, fat, and fibre content, while simultaneously reducing the total starch content. These mushrooms, rich in protein and polyunsaturated fatty acids, enhanced the nutritional value of the bread. Furthermore, the starch content in the bread decreased as the proportion of mushroom powder increased, while the dietary fibre content rose, which further contributed to a lower predicted glycaemic index of the bread [39,40,49].

### 3.7. Mineral Composition

The Reishi fruiting body powder exhibits significantly higher levels of calcium, iron, manganese, and copper compared to type 750 wheat flour, with increases of 268%, 620%, 864%, and 368%, respectively. The results of this study demonstrated a significant impact of Reishi powder addition on the macro- and microelement content of bread (Table 6). The Reishi powder contained less phosphorus and potassium compared to type 750 wheat flour, resulting in a reduction in these macroelements in the bread of approximately 29% for phosphorus and 22% for potassium. In contrast, the higher calcium content in Reishi relative to wheat flour translated into an increased calcium level in the enriched bread compared to the control sample. Similar results were obtained by incorporating Boletus edulis mushroom flour into bread, where the authors noted increased levels of calcium, magnesium, and iron [50]. The rise in calcium content in Reishi-enriched bread may be significant in the prevention of osteoporosis, degenerative joint diseases, and in conditions of increased calcium demand, such as menopause, periods of rapid growth, or for individuals engaged in intense physical activity [51]. Like *Boletus edulis*, *Pleurotus ostreatus*, and *Agaricus bisporus*, Reishi powder is notable for its high iron content (18.0 mg/100 g) and manganese (7.95 mg/100 g), which resulted in an increased presence of these elements in enriched bread (Table 6). In BR12, the iron content increased by approximately 32% compared to the control bread, which constitutes a significant improvement in nutritional value. An increased dietary intake of iron is particularly important in preventing anaemia, which often affects women of reproductive age, children, and the elderly. Iron plays a key role in oxygen transport within the body, and its deficiency can lead to weakness, fatigue, and impaired concentration [52,53]. The manganese content in BR12 bread increased by 61.9% compared to the control bread, which is crucial for proper skeletal system function, metabolism, and protection against oxidative stress. Increased manganese intake may support bone and joint health, as well as regenerative processes in the body [54].

### 3.8. Polyphenol Content and Antioxidant Activity

Polyphenols play a crucial role in antioxidant mechanisms, contributing to the neutralisation of free radicals and maintaining redox balance, which is a key element in protecting against oxidative damage. This mechanism is vital for cellular health and the overall well-being of the body [20]. Previous studies have shown that enriching bread with various raw materials rich in flavonoids and polyphenols, such as grape seed extract or prickly pear pulp, leads to increased antioxidant activity in the products [54]. The results of this study, presented in Table 7, confirm that Reishi powder is characterised by a significantly higher content of flavonoids (1.06 mg QE/g d.m.) and polyphenols (12.70 mg GAE/g d.m.) compared to wheat flour, in which no flavonoids were detected, and polyphenol content was only 0.13 mg GAE/g d.m. Additionally, Reishi powder exhibited very high antioxidant activity, achieving 98.5% in the ABTS·+ assay and 98.88% in the DPPH· assay, confirming its strong antioxidant properties. Substituting wheat flour with Reishi powder in bread led to a marked increase in the content of bioactive compounds and antioxidant activity. In the enriched bread (BR12 sample), flavonoid content was found to be 0.21 mg QE/g d.m., a significant increase compared to the control sample, where no flavonoids were detected. Polyphenol content increased more than fourfold, reaching 1.90 mg GAE/g d.m. Enriching bread with Reishi powder also resulted in a notable increase in antioxidant activity, with ABTS·+ radical scavenging rising by more than 160% (to 79.12%) and DPPH· radical scavenging increasing by over 45% (to 62.21%). Similar effects were observed in studies on gluten-free bread enriched with Chaga mushroom, where the content of flavonoids and polyphenols increased proportionally with the amount of the additive (from 5% to 20%), also leading to a significant increase in the bread’s antioxidant properties [43]. However, it was noted that higher doses of Chaga may negatively affect the sensory qualities of the bread, such as colour and texture, suggesting the need for further research to optimise the quantity of the additive to balance health benefits with the sensory acceptability of the product.

## 4. Conclusions

Studies on the enrichment of wheat bread with Reishi (*Ganoderma lucidum*) mushroom powder have shown that this addition significantly affects the rheological properties of dough, the physicochemical parameters of bread, and its nutritional value, while also offering considerable health benefits. The introduction of Reishi into wheat dough increased water absorption and extended dough stability time. This effect is attributed to the presence of polysaccharides, which have a high capacity for water binding and modify interactions within the gluten structure, leading to an extended dough development time and increased stability. The addition of Reishi also influenced the physical properties of the bread, such as a reduction in loaf volume, which resulted from the disruption of the gluten network by the fibre and polysaccharides present in Reishi. These components limited gluten’s ability to retain fermentation gases, resulting in a denser and less aerated crumb structure. However, the increased moisture content in the bread, due to the Reishi addition, may positively affect the bread’s texture and prolong its freshness. Nutritional analysis of Reishi-enriched bread revealed a significant increase in dietary fibre, protein, and essential minerals such as calcium, iron, and manganese. The addition of Reishi also enhanced the polyphenol content and antioxidant potential of the bread. Despite the undeniable benefits to the nutritional value of the bread, it is recommended that the amount of Reishi added to wheat bread should not exceed 6%, due to the negative impact of higher substitution levels on the sensory qualities of the products, particularly in terms of taste, aroma, and flexibility.

## Figures and Tables

**Table 1:** Farinographic parameters of dough.

Sample	DDT (min)	WA (%)	ST (min)	DS [FU]	FQN (mm)
CON	02:30 ^b^ ± 0.12	57.9 ^a^ ± 0.3	06:03 ^a^ ± 0.2	55.0 ^e^ ± 2.4	71.0 ^a^ ± 5.2
BR3	01:32 ^a^ ± 0.08	59.6 ^ab^ ± 0.2	09:23 ^b^ ± 0.2	35.0 ^d^ ± 1.8	66.0 ^a^ ± 2.8
BR6	01:48 ^a^ ± 0.1	60.8 ^ab^ ± 0.2	14:37 ^c^ ± 0.4	23.0 ^c^ ± 2.1	146.0 ^b^ ± 3.2
BR9	01:52 ^a^ ± 0.08	61.9 ^ab^ ± 0.5	15:00 ^c^ ± 0.2	17.0 ^b^ ± 1.2	172.0 ^c^ ± 3.4
BR12	10:48 ^c^ ± 0.3	63.3 ^b^ ± 0.2	19:08 ^d^ ± 0.3	1.0 ^a^ ± 0.8	207.0 ^d^ ± 4.1

CON—control sample (100% wheat flour); BR3, BR6, BR9, BR12—samples enriched with 3, 6, 9, and 12% of Reishi, respectively. Data are presented as mean (*n* = 3) ± standard deviation. Means in the column with different letters are significantly different (Tukey’s test; *p* ≤ 0.05). Tested parameters: DDT—development time; WA—water absorption; ST—dough stability; DS—degree of dough softening; FQN—farinograph quality number.

**Table 2:** Physical properties of bread.

Sample	Dough Yield [%]	Bread Yield [%]	Total Baking Loss [%]	Volume of 100 g of Bread
CON	162.17 ^a^ ± 0.5	140.78 ^a^ ± 1.31	13.19 ^c^ ± 0.81	350.79 ^e^ ± 2.69
BR3	162.67 ^ab^ ± 0.2	143.07 ^b^ ± 0.2	12.05 ^bc^ ± 0.3	337.27 ^d^ ± 2.1
BR6	162.83 ^ab^ ± 0.3	143.44 ^b^ ± 0.9	11.91 ^abc^ ± 0.7	329.35 ^c^ ± 2.2
BR9	163.50 ^b^ ± 0.3	146.48 ^c^ ± 0.4	10.28 ^ab^ ± 0.5	257.10 ^b^ ± 2.1
BR12	165.0 ^c^ ± 0.2	148.16 ^c^ ± 0.72	10.14 ^a^ ± 0.2	241.32 ^a^ ± 2.2

CON—control sample (100% wheat flour); BR—samples enriched with 3, 6, 9, and 12% of Reishi. Data are presented as mean (*n* = 3) ± standard deviation. Means in the column with different letters are significantly different (Tukey’s test; *p* ≤ 0.05).

**Table 3:** Colour of bread.

Simple	L*	a*	b*	Browning Index	Yellowness Index	Whiteness Index	ΔE*
CON	61.49 ^c^ ± 0.58	0.51 ^a^ ± 0.37	12.27 ^a^ ± 0.46	22.11 ^a^ ± 0.98	28.50 ^a^ ± 1.08	59.58 ^c^ ± 0.55	-
BR3	39.63 ^b^ ± 3.5	7.87 ^b^ ± 1.8	17.67 ^b^ ± 0.9	43.30 ^b^ ± 1.3	63.70 ^b^ ± 1.3	36.61 ^b^ ± 1.0	22.61 ^a^ ± 1.1
BR6	30.61 ^ab^ ± 1.7	8.00 ^b^ ± 0.9	15.71 ^b^ ± 0.8	46.13 ^c^ ± 1.2	73.32 ^c^ ± 1.2	28.41 ^a^ ± 1.1	30.86 ^b^ ± 1.2
BR9	25.89 ^a^ ± 1.8	9.09 ^b^ ± 1.7	13.28 ^a^ ± 0.8	47.20 ^c^ ± 1.4	73.28 ^c^ ± 1.4	24.16 ^a^ ± 1.3	35.55 ^c^ ± 1.3
BR12	22.60 ^a^ ± 1.6	9.29 ^b^ ± 1.2	10.54 ^a^ ± 0.7	46.46 ^c^ ± 1.3	66.63 ^bc^ ± 1.5	21.34 ^a^ ± 1.4	38.84 ^d^ ± 1.1

CON—control sample (100% wheat flour bread); BR—bread enriched with 3, 6, 9, and 12% Reishi. Colour parameters include L* (brightness), a* (shade from red to green), and b* (shade from blue to yellow). Data are presented as mean (*n* = 10) ± standard deviation. Mean values for the same parameters, denoted by different letters, are significantly different (Tukey’s test, *p* ≤ 0.05).

**Table 4:** Analysis of the texture profile of bread.

Sample	Hardness [N]		Springiness		Chewiness [N]		Cohesiveness		Crust Hardness [N]	
	24 h	48 h	24 h	48 h	24 h	48 h	24 h	48 h	24 h	48 h
CON	2.15 ^aA^ ± 0.25	2.26 ^aA^ ± 0.53	0.95 ^cA^ ± 0.05	0.96 ^dA^ ± 0.04	1.39 ^aA^ ± 0.10	1.36 ^aA^ ± 0.19	0.69 ^cA^ ± 0.03	0.64 ^cA^ ± 0.06	19.24 ^bB^ ± 1.53	15.64 ^abA^ ± 1.23
BR3	4.39 ^bA^ ± 0.25	4.70 ^bA^ ± 0.49	0.92 ^bcA^ ± 0.04	0.86 ^cdA^ ± 0.04	2.45 ^bA^ ± 0.22	2.29 ^bA^ ± 0.24	0.61 ^bA^ ± 0.03	0.56 ^bA^ ± 0.02	18.94 ^bB^ ± 1.10	14.98 ^aA^ ± 0.98
BR6	7.70 ^cA^ ± 0.93	12.90 ^cB^ ± 0.77	0.90 ^abcA^ ± 0.03	0.88 ^bA^ ± 0.03	3.99 ^cA^ ± 0.48	5.20 ^cB^ ± 0.37	0.58 ^bB^ ± 0.04	0.46 ^aA^ ± 0.01	18.18 ^abA^ ± 1.13	17.88 ^bA^ ± 1.64
BR9	22.03 ^dB^ ± 1.28	20.18 ^dA^ ± 1.84	0.88 ^abA^ ± 0.02	0.86 ^cdA^ ± 0.03	8.59 ^dA^ ± 0.5	7.54 ^dA^ ± 0.61	0.45 ^aA^ ± 0.02	0.44 ^aA^ ± 0.03	18.48 ^abA^ ± 1.28	17.68 ^bA^ ± 1.62
BR12	31.23 ^eA^ ± 2.38	30.38 ^eA^ ± 2.27	0.85 ^aA^ ± 0.03	0.82 ^aA^ ± 0.02	12.11 ^eA^ ± 0.65	11.66 ^eA^ ± 0.81	0.46 ^aA^ ± 0.02	0.47 ^aA^ ± 0.02	16.54 ^aA^ ± 0.97	18.12 ^bB^ ± 1.27

CON—control sample (100% wheat flour); BR—bread enriched with 3, 6, 9, and 12% Reishi. Data are presented as mean (*n* = 7) ± standard deviation. Different lowercase letters (a–e) in the column and different uppercase letters (A, B) in the row indicate statistically significant differences (Tukey’s test, *p* ≤ 0.05).

**Table 5:** The chemical composition of raw materials and bread.

Sample	Moisture	Ash	Protein	Crude Fat	TDF	IDF	SDF	Digestible Carbohydrate (CHO)	Calories [kcal/100 g]
	%	% d.m.							
Wheat flour type 750	9.4 ^A^ ± 0.1	0.69 ^A^ ± 0.01	8.79 ^A^ ± 0.04	0.45 ^A^ ± 0.03	5.3 ^A^ ± 0.13	2.4 ^A^ ± 0.1	2.9 ^A^ ± 0.2	71.04 ^B^ ± 0.03	351.30 ^B^ ± 0.11
Reishi powder	2.46 ^B^ ± 0.14	3.90 ^B^ ± 0.2	15.49 ^B^ ± 0.1	0.99 ^B^ ± 0.05	72.80 ^B^ ± 0.4	68.22 ^B^ ± 0.51	4.58 ^B^ ± 0.03	11.38 ^A^ ± 0.04	233.93 ^A^ ± 0.1
CON	41.46 ^a^ ± 0.71	2.20 ^a^ ± 0.01	10.83 ^a^ ± 0.04	1.23 ^a^ ± 0.03	7.21 ^a^ ± 0.19	4.26 ^a^ ± 0.17	2.95 ^a^ ± 0.02	78.52 ^e^ ± 0.42	224.16 ^d^ ± 0.17
BR3	44.18 ^ab^ ± 0.23	2.22 ^b^ ± 0.02	11.30 ^a^ ± 0.02	1.25 ^a^ ± 0.12	10.13 ^b^ ± 0.86	7.70 ^b^ ± 0.93	2.43 ^a^ ± 0.07	67.49 ^d^ ± 0.3	193.51 ^c^ ± 0.2
BR6	46.59 ^b^ ± 0.30	2.31 ^b^ ± 0.02	13.03 ^b^ ± 0.02	1.25 ^a^ ± 0.09	12.73 ^bc^ ± 1.35	10.07 ^b^ ± 1.32	2.66 ^b^ ± 0.03	63.21 ^c^ ± 0.24	182.48 ^b^ ± 0.5
BR9	45.17 ^c^ ± 0.2	2.35 ^c^ ± 0.05	13.37 ^b^ ± 0.05	1.25 ^a^ ± 0.1	14.49 ^c^ ± 0.02	11.81 ^b^ ± 0.03	2.68 ^b^ ± 0.01	61.07 ^b^ ± 0.2	185.32 ^ab^ ± 0.1
BR12	45.52 ^c^ ± 0.15	2.40 ^d^ ± 0.02	14.27 ^c^ ± 0.1	1.25 ^a^ ± 0.07	17.08 ^d^ ± 0.16	14.37 ^c^ ± 0.11	2.71 ^c^ ± 0.04	57.38 ^a^ ± 0.22	180.88 ^a^ ± 0.3

CON—control sample (100% wheat flour); BS—bread with 3, 6, 9, and 12% addition of Reishi; CHO—digestible carbohydrates (from difference); mean value (*n* = 3) ± SD; different letters (a–e) and (A, B) in the column indicate statistically significant differences (Tukey’s test, *p* ≤ 0.05). The lowercase letters (a–e) represent significant differences within a group of bread samples, while the uppercase letters (A, B) indicate significant differences between different groups of raw material samples, based on Tukey’s test (*p* ≤ 0.05).

**Table 6:** Content of selected minerals in raw materials and bread.

Sample	Macroelements			Microelements				Heavy Metals	
	P	Ca	K	Fe	Cu	Mn	Se	*Pb	*Cd
Content [mg/100 g]									
Wheat flour type 750	131 ^B^ ± 0.1	22.8 ^A^ ± 0.1	171.0 ^B^ ± 0.5	2.50 ^A^ ± 0.08	0.16 ^A^ ± 0.02	0.825 ^A^ ± 0.07	0.0112 ^A^ ± 0.0003	ND	ND
RE powder	93 ^A^ ± 0.2	84.0 ^B^ ± 0.2	133 ^A^ ± 11	18.0 ^B^ ± 0.12	0.75 ^B^ ± 0.5	7.95 ^B^ ± 0.18	0.0132 ^A^ ± 0.0005	ND	ND
CON	89.4 ^b^ ± 0.1	11.60 ^a^ ± 0.1	121 ^b^ ± 6	1.36 ^b^ ± 0.05	0.175 ^a^ ± 0.07	0.483 ^b^ ± 0.07	0.0081 ^c^ ± 0.0001	ND	ND
BR3	61.0 ^a^ ± 0.1	14.14 ^b^ ± 0.1	81.29 ^a^ ± 0.5	1.10 ^a^ ± 0.06	0.200 ^ab^ ± 0.10	0.455 ^a^ ± 0.02	0.0053 ^b^ ± 0.0002	ND	ND
BR6	61.0 ^a^ ± 0.1	15.93 ^c^ ± 0.1	80.6 ^a^ ± 0.2	1.38 ^b^ ± 0.08	0.210 ^abc^ ± 0.10	0.582 ^c^ ± 0.05	0.0063 ^a^ ± 0.0002	ND	ND
BR9	61.0 ^a^ ± 0.1	17.44 ^d^ ± 0.1	79.5 ^a^ ± 0.7	1.55 ^c^ ± 0.04	0.228 ^bc^ ± 0.20	0.691 ^d^ ± 0.02	0.0065 ^a^ ± 0.0003	ND	ND
BR12	61.0 ^a^ ± 0.1	19.44 ^e^ ± 0.1	79.66 ^a^ ± 0.6	1.8 ^d^ ± 0.02	0.246 ^c^ ± 0.10	0.782 ^e^ ± 0.03	0.0070 ^a^ ± 0.0004	ND	ND
RDA/AI [mg/day]	700	800	2000	14	1	2	0.055	0.428	0.06
% RDA/AI [%]									
CON	12.74	1.45	6.05	9.71	17.5	24.15	14.72	-	-
BR3	8.71	1.77	4.06	7.86	20	22.75	9.7	-	-
BR6	8.71	1.99	4.03	9.86	21	29.1	11.5	-	-
BR9	8.71	2.18	3.98	11.07	22.8	34.55	11.9	-	-
B12	8.71	2.43	3.98	12.86	24.6	39.1	12.72	-	-

RDA—recommended dietary allowances; AI—adequate intake; *daily allowable dose (WHO); CON—control sample (wheat bread); BR—bread enriched with 3, 6, 9, 12% Reishi (RE). Data are presented as mean (*n* = 3) ± standard deviation, means in the same column (raw material or sample) with different letters are significantly different (Tukey’s test; *p* ≤ 0.05).

**Table 7:** Polyphenol content and antioxidant activity of raw materials and loaves of bread.

Sample	Flavonoids (mg QE/g d.m.)	Polyphenols (mg GAE/g d.m.)	Radical Scavenging Activity against ABTS^·+^ (%)	Radical Scavenging Activity against DPPH^·^ (%)
Wheat flour type 750	ND	0.13 ^A^ ± 0.03	30.32 ^A^ ± 0.9	45.02 ^A^ ± 0.8
Reishi powder	1.06 ± 0.05	12.70 ^B^ ± 0.1	98.5 ^B^ ± 1.1	98.88 ^B^ ± 2.1
CON	ND	0.46 ^a^ ± 0.02	30.12 ^a^ ± 0.8	42.84 ^a^ ± 0.3
BR3	0.02 ^a^ ± 0.02	1.00 ^bc^ ± 0.08	42.51 ^b^ ± 0.12	53.12 ^b^ ± 0.8
BR6	0.03 ^a^ ± 0.01	1.10 ^c^ ± 0.05	54.38 ^c^ ± 0.42	56.42 ^c^ ± 1.1
BR9	0.10 ^b^ ± 0.01	1.26 ^c^ ± 0.02	61.91 ^d^ ± 0.5	59.90 ^d^ ± 0.2
BR12	0.21 ^b^ ± 0.02	1.90 ^d^ ± 0.1	79.12 ^e^ ± 1.0	62.21 ^e^ ± 0.3

CON—control sample (wheat bread); BS—bread with 3, 6, 9, and 12% addition Reishi. Data are presented as mean (*n* = 3) ± standard deviation; means in the same column (raw material or sample) with different letters are significantly different (Tukey’s test; *p* ≤ 0.05).

## Data Availability

The original contributions presented in the study are included in the article. Further inquiries can be directed to the corresponding author.
